# Association between Condylar Bone Changes and Eichner Index in Patients with Temporomandibular Dysfunction: A Cone Beam Computed Tomography Study

**DOI:** 10.30476/DENTJODS.2021.92488.1653

**Published:** 2023-03

**Authors:** Maryam Paknahad, Leila Khojastepour, Salma Tabatabaei, Mohammad Mahjoori-Ghasrodashti

**Affiliations:** 1 Oral and Dental Disease Research Center, Dept. of Oral and Maxillofacial Radiology, School of Dentistry, Shiraz University of Medical Sciences, Shiraz, Iran; 2 Dept. of Oral and Maxillofacial Radiology, School of Dentistry, Shiraz University of Medical Sciences, Shiraz, Iran; 3 Postgraduate Student, Eastman Institute for Oral Health, Rochester, United States

**Keywords:** Cone beam computed tomography, Eichner index, Temporomandibular joint, Temporomandibular Joint Disorders

## Abstract

**Statement of the Problem:**

Eichner index is a dental index, which is based on the occlusal contacts between naturally existing teeth in premolar and molar regions. One controversial topic is the association between occlusal status and temporomandibular joint dysfunction (TMD) and its associated degenerative bony changes.

**Purpose:**

Through the use of cone-beam computer tomography (CBCT), the current study sought to ascertain the relationship between the Eichner index and condylar bone alterations in TMD patients.

**Materials and Method:**

In this retrospective study, the CBCT images of bilateral temporomandibular joints (TMJs) of 107 patients with TMD were evaluated. The patients’ dentition was classified into three groups of A (71%), B (18.7%), and C (10.3%), according to the Eichner index. Radiographic indicators of condylar bone alterations, including as flattening, erosion, osteophytes, marginal sclerosis, subchondral sclerosis, and joint mice, were either present or absent and registered as 1 or 0, respectively. Chi-square test was used to evaluate the link between the condylar bony changes and the Eichner groups.

**Results:**

According to the Eichner index, the most prevalent group was group “A”. The most prevalent radiographic finding was “flattening of the condyles” (58%). Condylar bony changes were found to be statistically related to age (*p*= 0.00). However, no significant relationship was found between sex and condylar bony changes (*p*= 0.80). There was a significant relationship between the Eichner index and condylar bony changes (*p*= 0.05).

**Conclusion:**

Patients with greater loss of tooth supporting zones have more condylar bony changes.

## Introduction

The temporomandibular joint (TMJ), one of the body's most intricate articulations, has a wide range of anatomical and physiological characteristics [ 1 ]. Different factors can affect the articular surface of the condyle. Because of continuous stimulation, the TMJ constantly remodels [ 2 ]. Adaptive responses of bone are flattening of the bony surface or marginal sclerosis in response to loading forces that exceed the normal tolerances of joint tissues. When the ability of the joint to excessive loading forces through remodeling is surpassed, other degenerative changes include subchondral sclerosis, osteophyte, joint mice, and Ely's cyst take place. These changes in the condyle can predispose temporomandibular dysfunction (TMD) [ 2 - 4 ]. 

TMD describes a group of clinical complaints that affect the stomatognathic system, mainly the muscles of mastication [ 5 ]. TMD has a multifactorial etiology. It has been found that several risk factors are associated with the symptoms and signs of TMD [ 6 ]. A number of links have been reported between dental status and TMD. The loss of teeth, especially posterior teeth, can end up in an increase in the occlusal load on the remaining teeth and appears to disturb the function of TMJs in later stages [ 7 ]. A clinical examination alone cannot adequately assess the osseous and soft tissues of the TMJs [ 8 ]. Therefore, radiographic evaluation of the osseous structures is required to detect possible destructive changes in the TMJs in patients with TMD. Various radiographic modalities have been introduced to evaluate condylar morphology [ 9 ]. It is likely that CBCT, a newly developed imaging technique in dentistry, will be the most effective method of assessing the osseous morphology of TMJ joints. 

Developed by Karl Eichner, the Eichner index can be applied for epidemiological studies and it is one of the most widely used dental indices. This index is effective in establishing intermaxillary contacts and extending functional dental invalidity [ 10 ]. Using this index, the posterior teeth are divided into four support zones based on whether occlusal contact exists between the premolars and molars.

The relationship between Eichner index and bony changes in the condylar region has only been studied in a few studies [ 6 , 29 ]. In these studies, however, bony changes in the condyle were evaluated using two-dimensional images. Thus, the purpose of this study was to determine whether condylar bone changes are associated with Eichner index in TMD patients using CBCT.

## Materials and Method

In accordance with IR.SUM.DENTAL.REC.1399.0908, the present study was approved by the Institutional Research Committee. In this study, 107 patients with clinical signs and symptoms of TMD who needed further CBCT investigation were recruited from the archives of a private clinic and an oral and maxillofacial radiology department. There was a wide age range of 16 to 80 years for the patients (39.57±3.31 years). Exclusion criteria were patients with an established history of temporomandibular surgery, acute trauma, congenital abnormalities, musculoskeletal or neurological diseases, and any systemic diseases potentially affecting joint morphology.

### Tooth loss recordings

**Figure 1:**
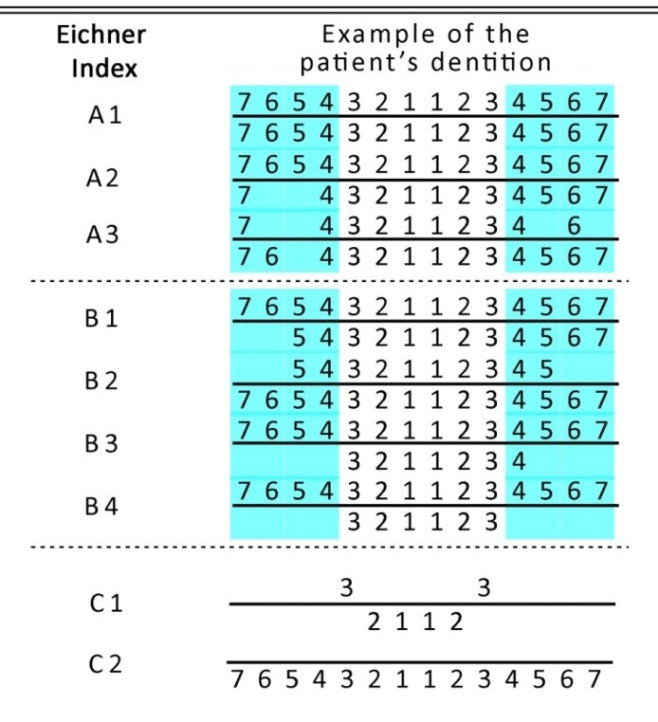
Classification by Eichner index

Based on the occluding pairs in the posterior teeth (two premolars and two molars), the dentition of each patient was divided into four main occlusal supporting zones. All of the four supporting zones are in contact in class A; one supporting zone is missing in class B, or all of the four supporting zones are absent, but the anterior region remains intact; and class C has no occlusal contact between the remaining teeth (Figure 1). This study considered both fully and partially erupted permanent teeth as "present teeth". Moreover, the supernumerary teeth, third molars, pontics of bridge prostheses, and implant-supported superstructures were not counted as the present teeth.

### CBCT of TMJs

A New Tom VGi (NewTom, Verona, Italy) was used to obtain CBCT images of bilateral TMJs with 110 KVp, 3.05 mA and 3.6 s exposure time in the standard resolution mode (voxel size 0.3). Image fields were 15×15cm. standing upright; the patients were biting their teeth in the maximum intercuspal position. The Frankfort plane was parallel to the floor when their heads were positioned. The NewTom Cone Beam 3D imaging system workstation (NNT Software version 6.2) was employed to prepare the images of TMJ. The reconstructed CBCT scans were assessed using high-resolution monitor (Barco-China) in a dedicated reporting room with appropriate viewing (dimly lit) condition.

The raw data were reconstructed primarily for the TMJ. By scrolling the axial images, the system identified the axial view on which the condylar width had the largest mediolateral dimension. The interval and thickness of the image slices were both set at 0.5mm. Afterwards, the corrected coronal and sagittal cross sections of each joint were rectified by drawing a perpendicular and parallel line and reconstructing them to the long axis of the condyle.

**Figure 2:**
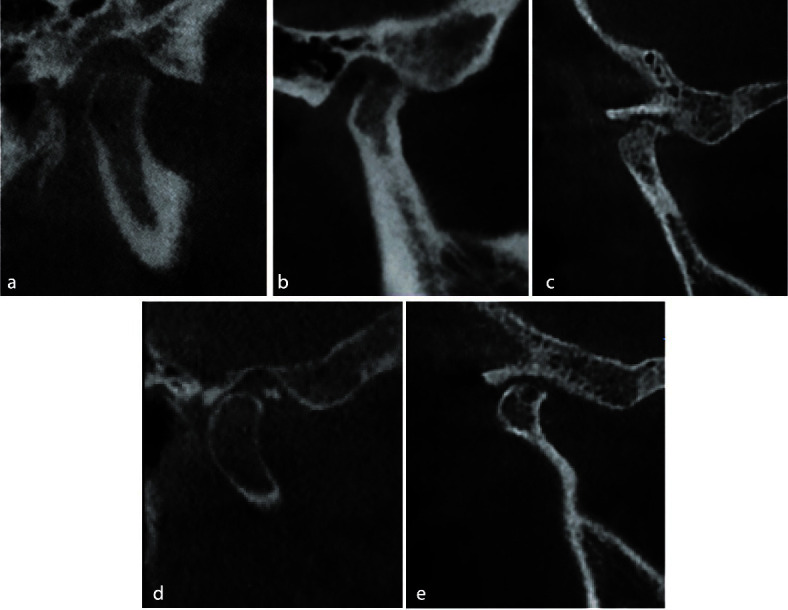
Condylar bone changes illustrated in sample images. These images have been taken from patients participated in the current study, **a:** Flattening of the condylar head, **b:** Surface erosion, **c:** Osteophyte, **d:** Loose joint body (joint mice), **e:** Subcortical cyst (Ely’s cyst)

The criteria used to evaluate condylar bony changes included (1) flattening (loss of convexity of condylar head outlines), (2) surface erosion (local area of rarefaction in the layer of compact bone), (3) marginal bony overgrowth or osteophytes (a local outgrowth of the bone arising from the mineralized surface), (4) subcortical erosion or Ely cyst (local area of rarefaction of the cancellous bone), (5) marginal sclerosis( thickening of the cortical bone), (6) subchondral sclerosis (Increased radiopacity of the cancellous bone), (7) joint mice (osteophytes that break off and lie free within the joint space) (Figure 2).

For each patient, the right and left TMJ areas were evaluated for the presence or absence of one or more of these radiographic changes, and were rated accordingly as 1 or 0.We calculated the left and right TMJ bony changes independently by adding up the scores related to any radiographic finding. For example, when a patient had flattening, erosion, and sclerosis in the condylar bones, the total number of changes was considered "3”. The right and the left scores were then added. Consequently, the association between the condylar bony changes and the Eichner index was examined.

### Measurement precision

The images were evaluated by two dentomaxillofacial radiologists. Each observer evaluated the images independently after a minimum of two weeks. In addition to checking the consistency between the first and the second sets of records produced by each specialist, we also examined the reliability of the inter-examiners for each of the criteria applying κ statistic. Adequate intra-examiner agreement index (0.831 to 1 and 0.833 to 1 for two specialists), as well as strong inter-examiner agreement (κ coefficient: 0.801 to 1) was detected.

### Statistical analyses

Statistical calculations were performed using SPSS (version 18, Chicago; IL, USA). To ensure the intra and inter-examiner reliability, κ statistic was used. The chi-square test was used to determine whether there was a correlation between condylar bony changes, age, sex, and the Eichner groups (A, B, and C). Significance level was set at *p*= 0.05. A Dunn’s post-hoc test was used to compare the prevalence of overall bone changes between Eichner groups. 

## Results

Excellent inter-(κ coefficient: 0.801 to 1) and intra-(κ c-oefficient: 0.833 to 1) examiner agreement was observe-d. In the present study, 107 patients were recruited among which 74 were females with an age range of 17-78 years (mean 39.18+15.20) and 33 were males with the age range of 16-80 years (mean 40.42+17.70). Based on the Eichner index, the most common Eichner group was a (71%) followed by group B (18.7%) and group C (10.3%) (Table 1).

**Table 1:** Distribution of Eichner index groups and gender

	Eichner index			Total (%)
	A	B	C	
Males	21(27.6%)	7(35%)	5(45.5%)	33(30.8%)
Females	55(72.4%)	13(65%)	6(54.5%)	74(69.2%)
Total	76(71%)	20(18.7%)	11(10.3%)	107

Out of the total 107 patients, 76 (71.02%) showed radiographic changes in condylar morphology. Flattening was the most prevalent bony changes (58.9%) followed by erosion (40.2%), marginal sclerosis (20.6%), subchondral sclerosis (14.0%), osteophyte (5.6 %), cyst (3.7%) and joint mice (0.9%). The results showed that total bony changes had a favorable relationship with age (*p*= 0.00). However, sex did not significantly affect total bony changes (*p*= 0.80). Bony changes in the condylar region were significantly associated with Eichner index (Table 2). Moreover, all bony changes, except those of joint mice showed significantly positive correlations with Eichner index. There was a statistically significant difference in the means of total bone changes across the groups (Table 3).

**Table 2:** The presence of various radiographic condylar bony change among Eichner groups

Radiographic Changes	Eichner Index				*p* Value*
	A	B	C	Total (%)	
Flattening	36(47.4%)	17(85%)	10(90%)	63(58.9%)	< 0.001
Erosion	19(25%)	15(75.5%)	9(81.8%)	43(40.2%)	< 0.001
Osteophyte	1(1.3)	2(10%)	3(27.3%)	6 (5.6%)	0.021
Cyst	0(0%)	1(50%)	3(27.3%)	4 (3.7%)	0.012
Marginal sclerosis	8(10.5%)	7(35%)	7(63.6%)	22(20.6%)	< 0.001
Subchondral sclerosis	2(2.6%)	6(20%)	7(63.6%)	15(14%)	< 0.001
Joint mice	0(0%)	0(0%)	1(6.1%)	1(0.9%)	0.100

* : Pearson’s Chi-square test

**Table 3:** An analysis of condylar bone changes in three groups based on the Eichner index

Eichner Index	N	Total bony changes		*p* Value*
		Mean±SD	Median	
A	76	1.19±1.22	1^A^	<0.001
B	20	3.75±1.91	4^B^	
C	11	6±1.89	5^C^	

* Median values with different upper case letters were statistically different (Dunn’s Post-hoc test)

## Discussion

A common cause of orofacial pain that is not related to dental or infectious conditions is TMD [ 12 ]. Several factors contribute to the symptoms and signs of TMD. One of the most controversial factors in dental literature is occlusion [ 13 - 15 ]. TMD has been associated with occlusion in some studies [ 16 - 20 ]. In other studies, however, no significant association between occlusion and TMD was found [ 6 , 14 , 21 - 23 ]. A definition-based debate between "occlusion" and "TMD" might explain the existing controversy, according to de Kanter *et al*. [ 24 ]. Many studies have evaluated the relationship between occlusion and TMD using various classifications of occlusion, including overjet, crossbite, and open bite [ 25 ]; canine class, molar class [ 13 ]; discrepancy between the centric relation and the intercuspal position [ 26 ], Kennedy classification, and Eichner index [ 2 , 6 , 29 ]. 

Since posterior teeth are necessary to maintain uniform occlusal force distribution on TMJ, losing them can have a greater impact than occlusion type. Additionally, due to incisors' inclined planes, entire mandible move backward when posterior teeth are lost. As a result of this movement, the condyles are moved above and behind their normal position in relation to the articular eminence. When a unilateral cause is responsible, only one condyle is affected but when the bite has a closed, both are affected [ 27 - 28 ].

Only two studies have investigated the relationship between Eichner index and condylar bony changes [ 6 , 29 ]. A significant correlation was found between Eichner index and condylar total bony changes in the present study. Findings of this study conflict with those of Takayama *et al*. [ 6 ] and Jalalian *et al*. [ 29 ]. According to Takayama *et al*. [ 6 ], despite the positive correlation between the Eichner index and the symptoms of TMD, A negative correlation was found between the Eichner index and the prevalence of bone changes in the condyle. The Eichner index and condylar bony changes were also not significantly correlated in a study by Jalalian *et al*. [ 29 ]. The variation in the findings in these studies can be explained by the limitations of the conventional radiographic modality used (panoramic radiography). Approximately 75% of condylar osteoarthritis changes detected by CT can be missed with panoramic radiography. Other reasons for the inconsistency of results of these studies with our study, besides imaging modality (panoramic radiography), include the time elapsed between tooth extractions and imaging time and oral habits, such as bruxism.

The TMJ is characterized by its ability to remodel when loading forces exceed its normal tolerance. In adults, this adaptive response can alter the condylar bone morphology and articular eminence [ 31 ]. Flattening of joint surfaces and sclerosis are the most common bony changes, which redistribute and resist loading forces over a larger surface [ 32 ]. Our study found that flattening is the most common bony change in the condylar region. Ahmed NF *et al*. [ 2 ], Mathew AL *et al*. [ 21 ], Gharge NR *et al*. [ 27 ] also found similar results. Many factors contribute to the prevalence of flattening as the most prominent bony change on condylar surfaces; it is the earliest alteration and bone change that occurs on articular surfaces in progressive diseases. It is thought that flattening is caused by the involvement of the masseter and temporal muscles, which overload the TMJ [ 12 ]. Unlike this study, Jalalian *et al*. [ 29 ] and Takayama *et al*. [ 6 ] found that Ely's cyst is most frequently seen on condylar radiographs. 

In the published literature, there is still controversy over the correlation between condylar bony changes and age. According to several studies, condylar bony changes and aging are positively correlated [ 4 , 29 , 34 - 35 ], while Takayama *et al*. [ 6 ] and Jalalian *et al*. [ 29 ] found no correlation. According to our study, total bony changes increase with age. Biological alterations and collective exposure to different risk factors may explain the rise in prevalence of total bony changes and their severity with age. Among these risk factors are weak muscle strength, decreased proprioception, oxidative injury, and cartilage thinning [ 15 ].

Our study found no significant relationship between sex and total bony changes, which is consistent with Irsan NDH *et al*.'s study [ 36 ]. Alzaharani *et al*. [ 35 ], Takayama *et al*. [ 6 ], and Jalalian *et al*. [ 29 ] reported a similar result. This is contrary to the findings of Gharge NR *et al*. [ 27 ], who found a higher prevalence of condylar bone alteration in women.

Various radiographic modalities such as plain film radiography [ 27 , 29 ], conventional tomography [ 37 ], computed tomography (CT) [ 38 - 39 ], CBCT [ 4 , 40 ], and magnetic resonance imaging (MRI) [ 41 - 42 ] have been used in previous studies to evaluate condylar morphology. In conventional radiography, such as panoramic radiography, the most lateral portion of condyle is represented as a 2-dimensional image. Using these radiographs to assess condylar morphology is therefore less reliable. The 1-3 mm thickness of conventional slices limits the accuracy of assessing changes in condylar morphology. MRI is a method used to assess the soft tissue parts of the TMJ. The use of CT is widely used in medicine as a diagnostic tool, but its availability, cost, and radiation exposure limit its use in dentistry. In comparison with spiral CT, CBCT has a sub-millimeter spatial resolution that is as high as or higher than spiral CT [ 43 ]. Therefore, CBCT was chosen as the preferred imaging modality.

This study aimed to determine whether radiographic bone changes in the condyles are associated with Eichner; however, some factors such as the time between tooth extraction and image time and oral habits were not evaluated in relation to the severity of TMD symptoms and condylar bony changes. To either confirm or refute the results of the present study, further investigation should be conducted with these factors taken into account.

## Conclusion

The results of this study indicate that condylar bony changes are highly associated with the Eichner index. In addition, CBCT findings were associated with variations in finding condylar bony changes in this study. It is therefore important to consider CBCT as a modality for appropriate diagnosis in clinical practice as well as for patients' therapeutic choices.

## Conflict of Interest

The authors declare that they have no conflict of interest.
